# The SLC6A19 gene mutation in a young man with hyperglycinuria and nephrolithiasis: a case report and literature review

**DOI:** 10.1186/s12894-022-01147-9

**Published:** 2022-11-24

**Authors:** Yang Pan, Shangren Wang, Li Liu, Xiaoqiang Liu

**Affiliations:** Department of Urology, Tianjin Medical University General Hospital, No. 154 Road Anshan, Heping District, 30052 Tianjin, China

**Keywords:** Hyperglycinuria, Nephrolithiasis, Nutcracker syndrome, SLC6A19, Case report

## Abstract

**Background:**

Hyperglycinuria is a rare disorder, with few reported cases, caused by either a defect in glycine metabolism or a disturbance in renal glycine reabsorption. Genetic findings of hyperglycinuria are rare and have not previously been reported in Chinese young men.

**Case presentation:**

A 24-year-old man presented with a compliant of bilateral lumbago for 1 month. Abdominal computed tomography revealed bilateral kidney stones and right upper ureteral dilatation. The 24-h urine analysis showed high urine oxalate levels of 63 mg/day. Analysis of amino acids in urine revealed that his urinary glycine levels were abnormally high (2.38 µmol/mg creatinine). Whole-exome sequencing detected the SLC6A19 variant c.1278 C > T p. (Cys426). Flexible ureteroscopy with holmium laser lithotripsy was conducted twice to remove his bilateral nephrolithiasis. Postoperative stone biochemical composition analysis revealed that the stones were composed of approximately 70% calcium oxalate monohydrate and 30% calcium oxalate dihydrate. The patient was subsequently diagnosed with hyperglycinuria. Three months after the stone surgery, ultrasonography revealed one nodule under the right thyroid lobe during a health checkup. His serum parathyroid hormone (PTH) levels increased to 392.3 pg/mL. Resection of the right parathyroid nodule was performed, and the histopathological examination confirmed right parathyroid adenoma. During the 2-year follow-up period, nephrolithiasis did not relapse, and serum PTH, calcium, and phosphorus levels were normal.

**Conclusion:**

The SLC6A19 gene may have been significant in the development of hyperglycinuria in a Chinese young man. Further evaluation for the possibility of a glycine excretion disorder could be considered when encountering nephrolithiasis.

## Background

Hyperglycinuria is an unusual disease owing to abnormal glycine metabolism or renal glycine reabsorption defect [1]. To date, there is little research and few case reports published on hyperglycinuria. Glycinuria has usually been a feature of other syndromes in which amino acids are excreted in the urine, such as Fanconi syndrome [2]. Genetic findings of hyperglycinuria are rare and have not previously been reported in Chinese young men. Hyperglycinuria is commonly shown as either sole hyperglycinuria or involvement of multiple aminoacidurias relevant to proline and hydroxyproline excretion [3]. Although the underlying cause of congenital disorders associated with amino acid transport may be benign, hyperglycinuria usually has extensive characteristics related to kidney stones [4].

In the present study, we report a rare case of hyperglycinuria combined with nephrolithiasis in which a mutation in the SLC6A19 gene was detected by genetic analysis. To our knowledge, this is the first report of hyperglycinuria with an SLC6A19 gene mutation in a Chinese young man. We further present a review of the relevant literature. We hope that this study will contribute to a better understanding of this diseases in the future.

## Case presentation

A 24-year-old Chinese man presented to our hospital with the primary complaint of bilateral lumbago for 1 month. The patient had no hematuria, fever, or other obvious symptoms. Mild pain on percussion was observed at the bilateral costovertebral angles, but no other significant findings on physical examination were detected. The patient did not have metabolic syndromes, such as obesity, diabetes mellitus, or hypertension. Moreover, his family and psycho-social history showed no obvious abnormality.

**Fig. 1:**
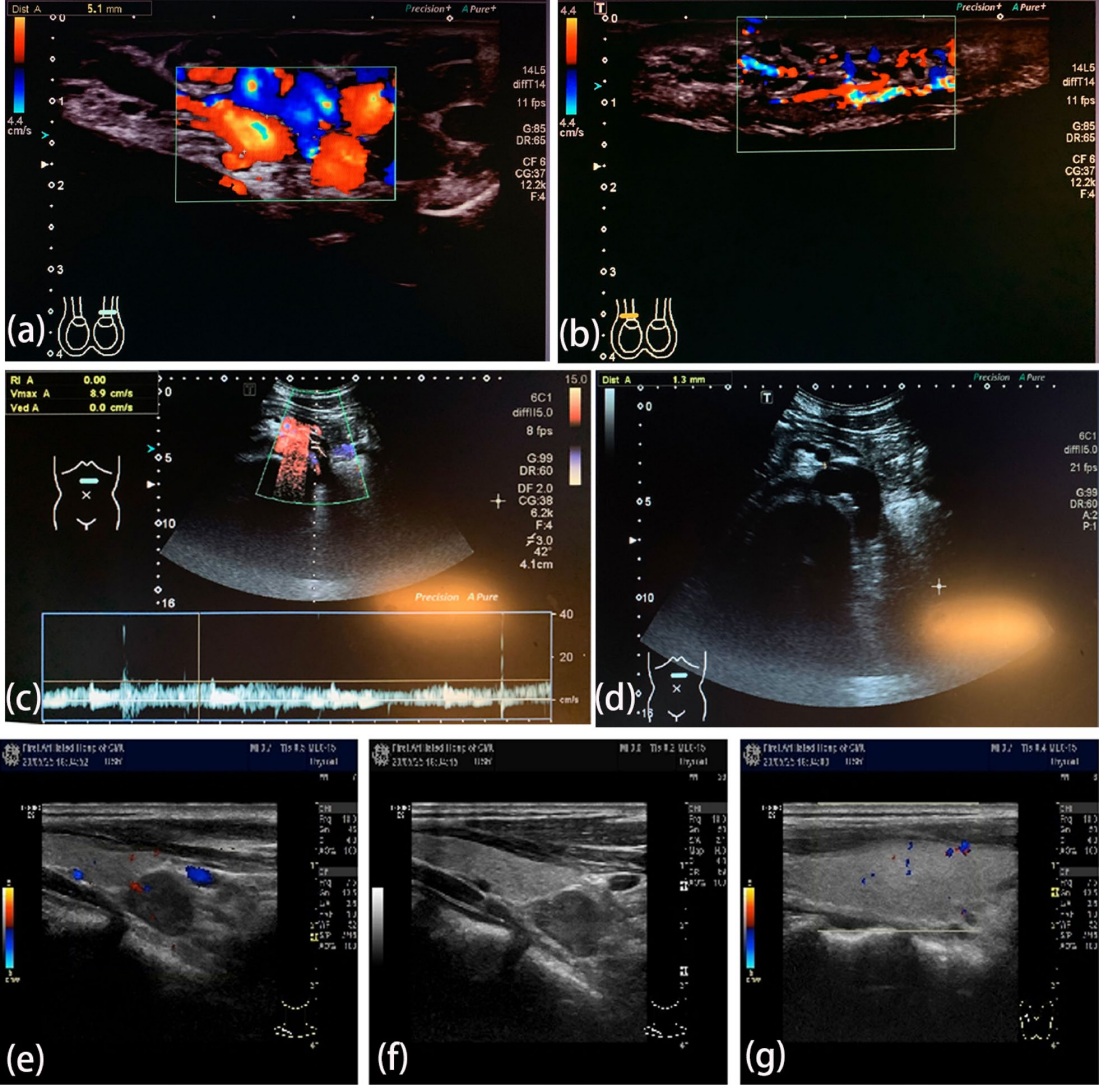
Ultrasonography revealed the left varicocele, nutcracker phenomenon, and right parathyroid nodule. **a** The left varicocele. **b** The right normal spermatic vein. **c**, **d** The left renal vein was compressed across the abdominal aorta. **e**, **f**, **g** Neck ultrasonography showed one hypoechoic nodule under the right lobe of the thyroid

**Fig. 2:**
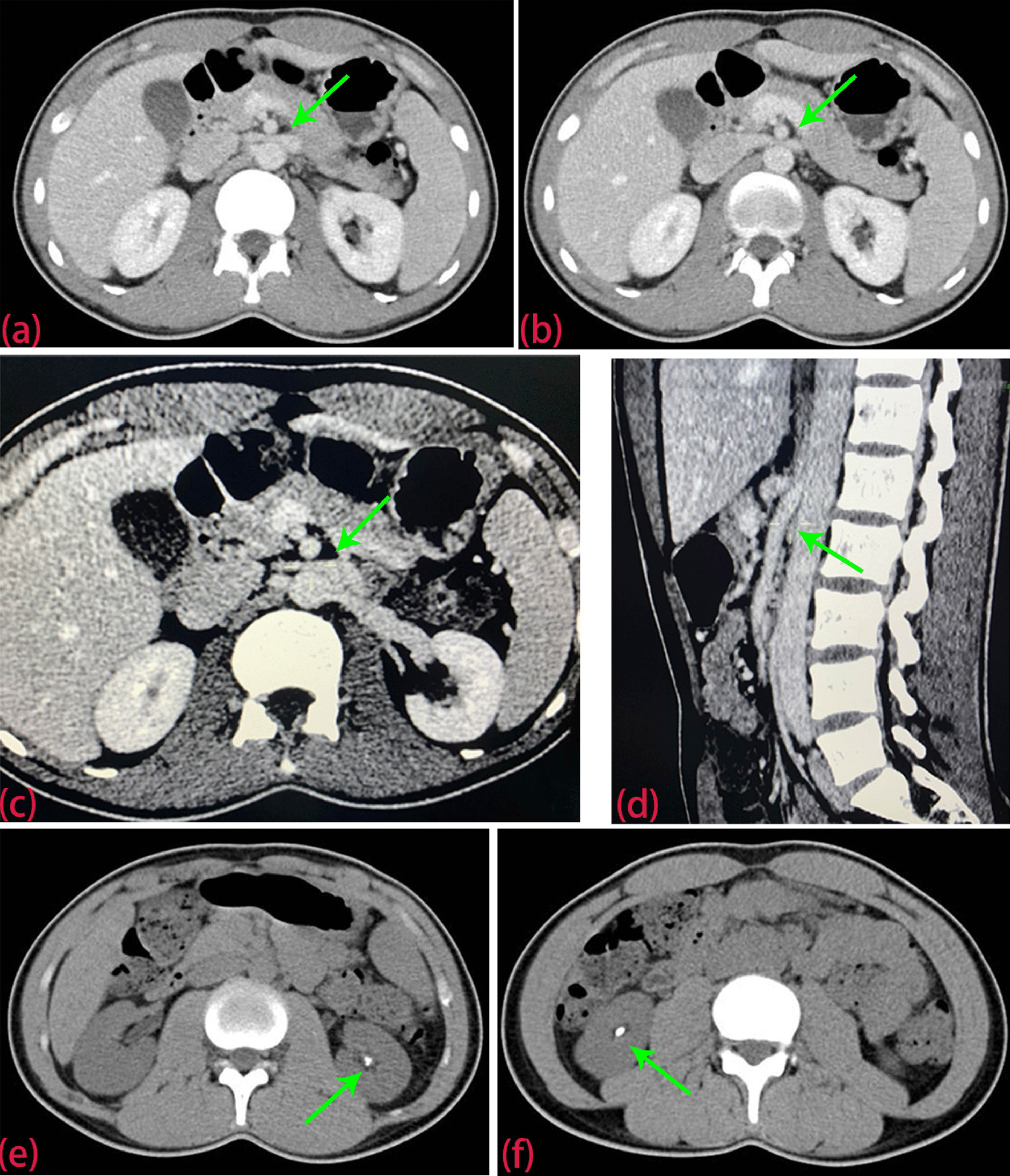
Computed tomography (CT) revealed the nutcracker phenomenon and bilateral kidney stones. **a**, **b**, **c**, **d** Abdominal CT angiography revealed that the angle between the left renal vein and the aorta had become smaller, showing the compression of left renal vein between superior mesenteric artery and abdominal aorta. **e** The left kidney stone. **f** The right kidney stone

Six months prior, the patient had developed scrotal pain after a strenuous activity. Ultrasonography revealed a left varicocele (Fig. 1a, b) and compression of the left renal vein across the abdominal aorta. These findings were consistent with the nutcracker phenomenon (Fig. 1c, d). Abdominal computed tomography (CT) angiography further revealed that the angle between the left renal vein and the aorta had become smaller, showing compression of the left renal vein between the superior mesenteric artery and the aorta (Fig. 2a–d). Therefore, the patient was diagnosed with nutcracker syndrome. Selective ligation of the left spermatic vein was performed. The operation was successful, and the varicocele was completely ligated.

**Fig. 3:**
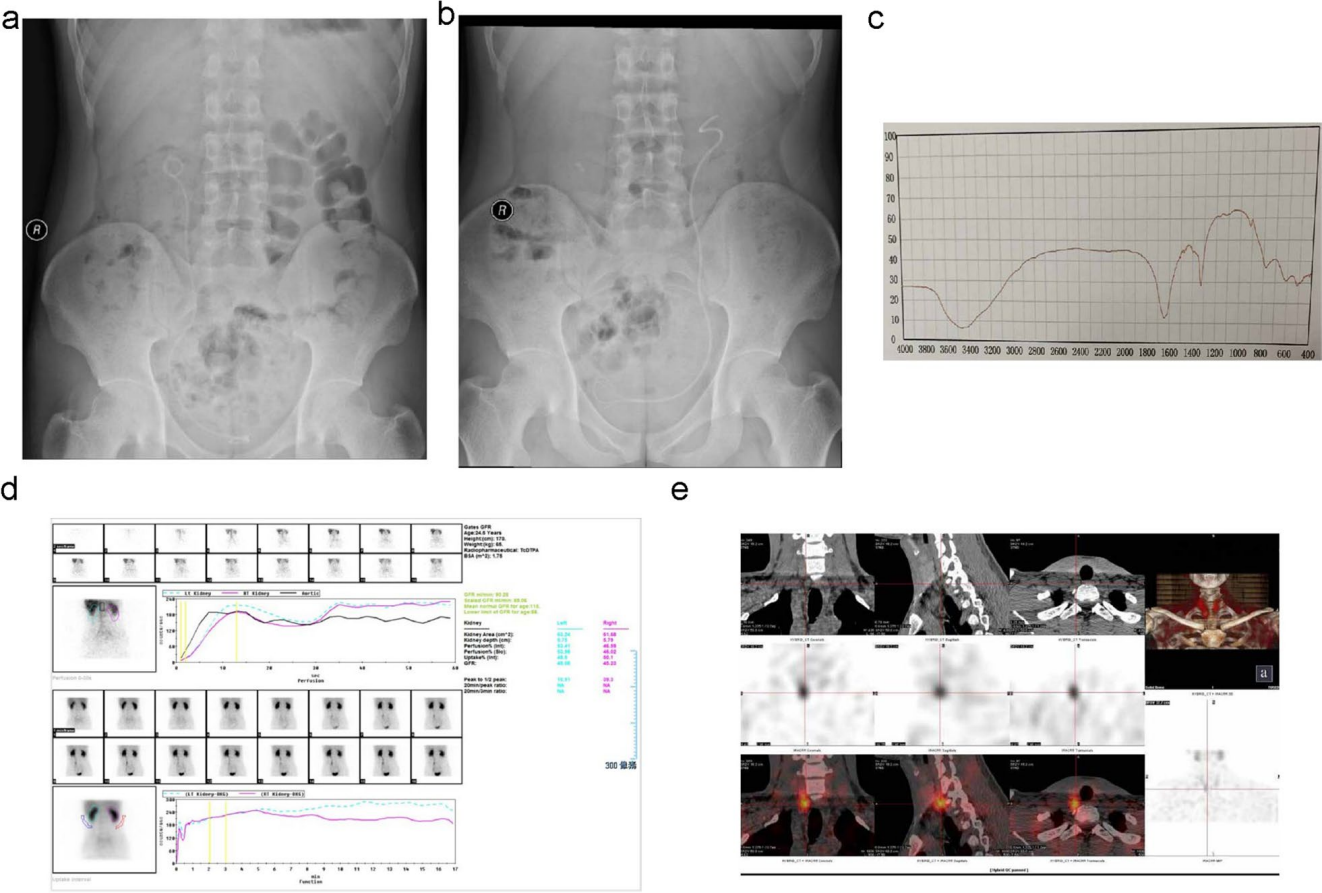
Abdominal X-ray of kidney, ureter, bladder (KUB) after the first (**a**) and second (**b**) flexible ureteroscopy lithotripsy. **c** Biochemical composition analysis of the stones was performed using the LIIR-20 type infrared spectrum automatic analysis system. **d** Single-photon emission computed tomography (SPECT) of bilateral kidneys revealed that glomerular filtration rates decreased slightly. **e** Neck SPECT revealed a round soft-tissue density shadow at the posterior radioactive concentration shadow of the lower right lobe of the thyroid, with a size of 15.8 mm × 8.8 mm and a clear boundary

When he was admitted into our hospital for bilateral lumbago this time, abdominal and pelvic CT scan revealed right upper ureteral dilatation with hydronephrosis and bilateral kidney stones (Fig. 2e–f). The left and right kidney stones were 1.1 and 1.6 cm in diameter, respectively, according to measurements on the CT scan. Single-photon emission computed tomography (SPECT) using diethylenetriaminepentaacetic acid tagged with ^99m^Tc (^99m^Tc-DTPA) was performed to evaluate the split renal function in this patient. SPECT revealed that the glomerular filtration rates decreased slightly to 45.05 and 45.23 mL/min in the left and right kidneys, respectively (Fig. 3d). Routine laboratory tests such as routine blood tests showed no obvious abnormalities. Twenty-four-hours urine analysis showed high urine oxalate levels of 63 mg/day (normal < 40 mg/day), while the urine calcium, phosphate, citrate, and uric acid levels were within normal range. To determine the possible cause for the bilateral nephrolithiasis and high urine oxalate levels in this patient, a urine amino acid screen was performed, which revealed urine glycine levels of 2.38 µmol/mg creatinine (normal 0.38–1.59 µmol/mg creatinine).

To identify possible genetic mutations, molecular analyses using whole-exome sequencing were performed in this patient. After obtaining informed consent from the patient, genomic DNA was extracted from the peripheral blood leukocytes by standard phenol-chloroform procedures. Whole-exome sequencing detected the SLC6A19 variant c.1278 C > T p. (Cys426), which was further confirmed using Sanger sequencing. Segregation analysis performed to detect mutations in his parents did not reveal the presence of the same variant.

To remove the bilateral nephrolithiasis, flexible ureteroscopy with holmium laser lithotripsy (f-URS) was successfully conducted twice. Abdominal X-ray of the kidney, ureter, and bladder (KUB) after the first and second f-URS showed no residual stones (Fig. 3a–b). The biochemical composition analysis of the stones was performed postoperatively using an LIIR-20 type infrared spectrum automatic analysis system (Tianjin Lanmod Scientific Instrument Co. Ltd, Tianjin, China), which revealed that the stones in this patient were composed of approximately 70% calcium oxalate monohydrate and 30% calcium oxalate dihydrate (Fig. 3c). This information was considered one of the significant pieces of evidence for the diagnosis of hyperglycinuria because glycine had played an important role in oxalate metabolism and could increase oxalate levels. Therefore, the patient was diagnosed with hyperglycinuria based on the typical symptoms (nephrolithiasis), laboratory testing (high urine glycine and oxalate levels), stone biochemical composition analysis (calcium oxalate), and relevant gene mutation (SLC6A19). After discharge, the patient did not receive special drugs and he was required to avoid a high-oxalate diet. A metabolic evaluation was performed one month after the procedure by collecting and analyzing serum samples and 24-h urine sample. Results of the serological tests including serum ionized calcium, phosphate, creatinine, and uric acid were normal. The 24-h urine analysis results, including urine calcium, phosphate, oxalate, citrate, and uric acid levels, were also within normal range.

**Fig. 4:**
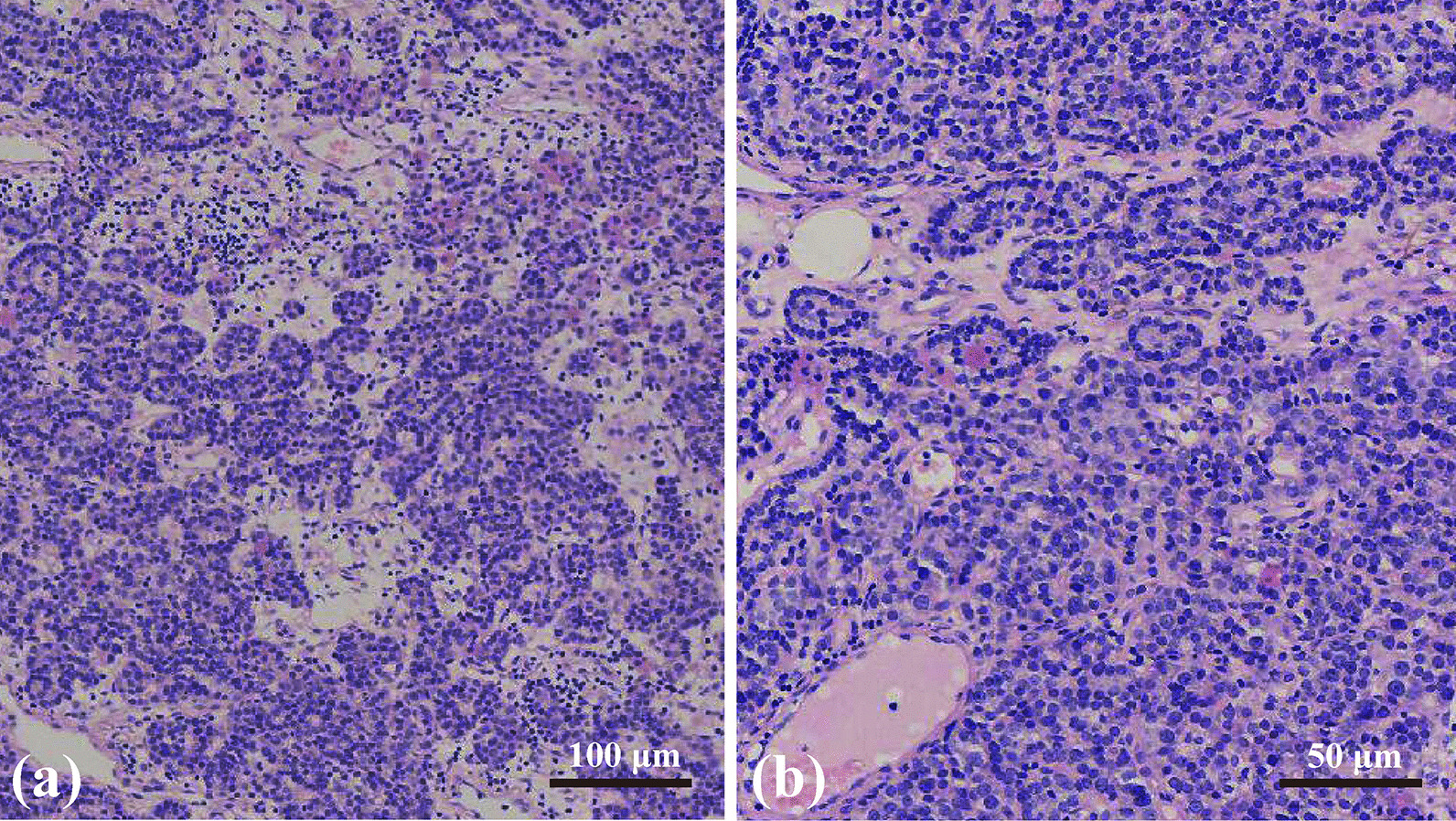
The hematoxylin and eosin (H&E) stained tissue sections revealed the typical pathological characteristics of parathyroid adenoma under the Olympus CX31 biological microscope. The capsule can be seen in the tumor tissue. There was a small amount of compressed residual parathyroid tissue outside the capsule. The tumor parenchyma was mainly composed of chief cells. Tumor cells were arranged in clumps, cords, or acinar-like structures. Large and scattered eosinophils can be seen in the tumor tissue. The cytoplasm of these eosinophils was stained red. **a** H&E stain, magnification 100×; **b** H&E stain, magnification 200×

Three months following the nephrolithiasis surgery, one nodule under the right lobe of the thyroid was detected on ultrasonography during a health checkup of the patient. Neck ultrasonography showed a hypoechoic nodule under the right lobe of his thyroid with a size of approximately 16 × 9 mm (Fig. 1e–g). Neck SPECT revealed a round soft-tissue density shadow at the posterior radioactive concentration shadow of the lower right lobe of the thyroid, with a size of approximately 15.8 × 8.8 mm and a clear boundary (Fig. 3e). Furthermore, his serum parathyroid hormone (PTH) levels increased to 392.3 pg/mL (normal < 70 pg/mL), while calcium levels in the blood and urine were normal. Therefore, he was considered for a diagnosis of adenomatous hyperparathyroidism. Resection of the right parathyroid nodule was performed under general anesthesia. His PTH levels normalized on the first day postoperatively. Postoperative hematoxylin and eosin-stained sections of excised tissue samples showed typical pathological characteristics of parathyroid adenoma (Fig. 4). During the 2-year follow-up period, the patient did not experience relapse of nephrolithiasis, and the levels of serum PTH, calcium, and phosphorus were maintained at the respective normal values. The patient was extremely satisfied with his entire treatment and follow-up process.

## Discussion and conclusions

In the present study, we report the case of a patient with bilateral nephrolithiasis, who was subsequently diagnosed with hyperglycinuria, left varicocele, nutcracker syndrome, and right parathyroid adenoma. Based on the important role of genes in disease development, we also analyzed and identified a mutation in the SLC6A19 gene. Our study showed that gene mutations may be a crucial factor in the development of hyperglycinuria and further emphasizes the importance of the timely detection, management, and prevention of hyperglycinuria.

The description of nutcracker syndrome was presented in our report because it was a relatively rare disease and was deemed an important aspect of the past medical history of this patient. After searching for the relevant literature and conducting bioinformatics analysis, we could not identify a common genetic pathway between nutcracker syndrome and hyperglycinuria. Hence, we consider that the coexistence of these two diseases in this patient might be a coincidence. Nutcracker syndrome can be classified as either anterior or posterior. Diagnosis of nutcracker syndrome is challenging due to the absence of definite and uniform criteria [5]. The gold standard way for diagnosing nutcracker syndrome is to measure the renal venous pressure by venography; however, this is an invasive method for patients. Doppler ultrasonography is regarded as a helpful and convenient imaging examination for nutcracker syndrome. Both CT and magnetic resonance imaging can reveal collateral circulation in the renal hilum, premature opacification of the left gonadal vein (suggestive of reflux), and reduction of the aortomesenteric angle (< 10º) [6]. The management of nutcracker syndrome is mainly based on patient age, symptom severity, and expectation for symptom reversal. When the patients are young and symptoms are not obvious, observational or symptomatic treatment is the preferred option. Endovascular surgical procedures or open surgery can be an alternative in cases with severe symptoms. The patient in our report had nutcracker syndrome; however, he did not develop recurrent varicocele or other symptoms such as hematuria or proteinuria. Moreover, the patient was relatively young. Therefore, we did not further treat his nutcracker syndrome and asked him to undergo regular follow-up.

Hyperparathyroidism can present as a solitary disease, or as a part of a syndrome. Germline mutations in the RET, CDC73, and CASR genes have been detected in greater than 10% of patients with hyperparathyroidism [7]. Among patients with multiple endocrine neoplasia type 2 A hyperparathyroidism, 97% showed gene mutations. Such individuals have an increased risk for development of parathyroid adenoma or hyperplasia, medullary carcinoma of the thyroid, and pheochromocytoma [8]. Furthermore, patients with somatic genetic mutations may develop sporadic parathyroid carcinoma. Therefore, patients with hyperparathyroidism could be counseled on germline genetic mutation examination.

In patients with kidney stones, conservative treatment can be applied for smaller stones. Fabris et al. reported that the use of potassium citrate showed good efficacy with a 48% decrease in the calcium levels and a 75% increase in the citrate levels [9]. Some lifestyle changes, such as increasing the intake of water, fruits, and vegetables, may also be helpful. However, if the stones are relatively large, minimally invasive procedures such as f-URS and percutaneous nephrolithotripsy can be used as alternatives [10]. Bilateral nephrolithiasis in this patient were relatively large based on imaging examinations. Thus, f-URS was performed to remove his nephrolithiasis.

Hyperglycinuria usually results from a defect in glycine metabolism or a disturbance in renal tubular reabsorption of glycine [4]. In some patients, factors such as high-glycine diet or total parenteral nutrition can increase glycine levels in the blood. When the elevated blood glycine levels exceed the reabsorption capacity of the renal tubules, the urine glycine levels can subsequently increase, thereby leading to hyperglycinuria. In other patients, a genetic defect or other unknown reasons can trigger a disturbance in renal tubular reabsorption of glycine, which can increase the glycine levels in the urine and cause hyperglycinuria [11].

Glycine plays an important role in oxalate metabolism and may result in an increase in oxalate levels [12]. Excess glycine can directly transform into glyoxylate by oxidative deamination or the serine-glycolate pathway [4]. Oxalate is formed as an end product of glyoxylate metabolism [13]. Thus, the excess glycine can be converted into oxalate following liver metabolism. Oxalate is secreted by glomerular filtration in the proximal nephrons. Urinary oxalate density can increase up to 40% due to continuous abundance from the glycine pool [14]. The increased oxalate levels in the urine can further promote calcium oxalate stones formation.

However, one recent study reported that high urine glycine levels may also play a potential role in preventing urine oxalate formation. In the study, Lan et al. [15] found that urine glycine levels in patients with calcium oxalate kidney stones were significantly lower than those in healthy people. Furthermore, they revealed that glycine could significantly attenuate ethylene glycol-induced calcium oxalate crystal depositions in the rat kidney by decreasing urine oxalate. Their finding might be different from previous view that glycine could increase oxalate. Consequently, we considered that glycine metabolism was relatively complex in human beings [16]. The conversion of glycine to oxalate may be reversible and depend on many factors.

Some previous reports revealed that the patients with hyperglycinuria were usually complicated by calcium oxalate nephrolithiasis [4, 13]. The patient in the present report was also consistent with this clinical manifestation. Therefore, we speculated that the typical symptom and stone composition in patients with hyperglycinuria may be nephrolithiasis and calcium oxalate, respectively. The formation of nephrolithiasis is a complex process associated with multiple causes. The combination of hyperglycinuria and calcium oxalate nephrolithiasis may indicate a potential link between the two, and relevant gene mutations could be significant.

The SLC6A19 gene can code the amino acid transporter B0AT1, which is crucial for transporting amino acids in the liver, kidney, and intestine [17]. It is well-known that the glomeruli in the renal cortex function to produce an ultrafiltrate. B0AT1 contributes to the reabsorption of amino acids from the ultrafiltration to the blood. Therefore, mutations in the SLC6A19 gene may result in a disturbance in renal reabsorption of amino acids and possible defects in the transportation of relevant metabolites. Disturbance in renal reabsorption of glycine can result in hyperglycinuria. Moreover, a defect in oxalate transportation may increase the urine oxalate content and trigger the damage to the renal tubular epithelial cells, which in turn could promote the formation of calcium oxalate stones [17]. The patient in our report did not receive a high-glycine diet or total parenteral nutrition. However, his urine glycine and oxalate levels were elevated. Thus, we considered that hyperglycinuria and calcium oxalate nephrolithiasis in this patient might mainly result from a disturbance in renal reabsorption of glycine and a defect in oxalate metabolism due to the SLC6A19 gene mutation. Nevertheless, the exact association and mechanism need to be explored and demonstrated in more patients in the future.

The treatment of diseases associated with the B0AT1 transporter or SLC6A19 gene is controversial. Some experts assume that pharmacological inhibition of the B0AT1 transporter or the SLC6A19 gene can be regarded as a method to manage amino acid imbalance with few side effects [18, 19]. The main mechanism of action of the inhibitors depends on the concurrent inhibition of amino acids in the kidneys and intestine in the presence of competing substrates [20, 21]. Javed et al. [22] attempted to predict the inhibition of SLC6A19 using biomarkers in biological samples, such as urine, plasma, and feces. They found that these biomarkers could successfully assess transporter inhibition in a mouse model. Their findings indicated that metabolite biomarkers could be used to confirm the inhibition of the corresponding transporters in the intestine and kidneys. Meanwhile, this also provide significant information on the management of diseases associated with SLC6A19 gene mutations.

The patient in our report had hyperglycinuria combined with left varicocele, bilateral nephrolithiasis, nutcracker syndrome, and parathyroid adenoma. The co-occurrence of these diseases in one patient is relatively rare. Furthermore, we found a relevant mutation in the SLC6A19 gene and possible genetic mechanisms of its association with hyperglycinuria. Therefore, this report is unique because the case was rare with many concurrent diseases in one patient, and it is the first report of the SLC6A19 gene mutation in a Chinese young man with hyperglycinuria.

In conclusion, this article describes the first case of a Chinese young man with hyperglycinuria and nephrolithiasis. It appears that the SLC6A19 gene mutation may have played a significant role in the development of hyperglycinuria in this patient. Further evaluation for the possibility of a glycine excretion disorder could be considered when encountering nephrolithiasis.

## Data Availability

The datasets used during the current study are available from the corresponding author on request.

## Abbreviations

CT: Computed tomography
SPECT: Single-photon emission computed tomography
f-URS: Flexible ureteroscopy with holmium laser lithotripsy
PTH: Parathyroid hormone
